# Comparing polysomnography, actigraphy, and sleep diary in the home environment: The Study of Women’s Health Across the Nation (SWAN) Sleep Study

**DOI:** 10.1093/sleepadvances/zpac001

**Published:** 2022-02-19

**Authors:** H Matthew Lehrer, Zhigang Yao, Robert T Krafty, Marissa A Evans, Daniel J Buysse, Howard M Kravitz, Karen A Matthews, Ellen B Gold, Sioban D Harlow, Laura B Samuelsson, Martica H Hall

**Affiliations:** Department of Psychiatry, University of Pittsburgh, Pittsburgh, PA, USA; Department of Statistics and Applied Probability, National University of Singapore, Singapore, Singapore; Deparment of Biostatistics and Bioinformatics, Emory University, Atlanta, GA, USA; Department of Psychology, University of Pittsburgh, Pittsburgh, PA, USA; Department of Psychiatry, University of Pittsburgh, Pittsburgh, PA, USA; Department of Psychiatry and Behavioral Sciences, Rush University Medical Center, Chicago, IL, USA; Department of Preventive Medicine, Rush University Medical Center, Chicago, IL, USA; Department of Psychiatry, University of Pittsburgh, Pittsburgh, PA, USA; Department of Public Health Sciences, University of California, Davis School of Medicine, Davis, CA, USA; Department of Epidemiology, University of Michigan, Ann Arbor, MI, USA; Department of Psychology, University of Pittsburgh, Pittsburgh, PA, USA; Department of Psychiatry, University of Pittsburgh, Pittsburgh, PA, USA

**Keywords:** sleep, methods, polysomnography, actigraphy, self-report, sleep diary, middle-aged women

## Abstract

**Study Objectives:**

Polysomnography (PSG) is considered the “gold standard” for assessing sleep, but cost and burden limit its use. Although wrist actigraphy and self-report diaries are feasible alternatives to PSG, few studies have compared all three modalities concurrently across multiple nights in the home to assess their relative validity across multiple sleep outcomes. This study compared sleep duration and continuity measured by PSG, actigraphy, and sleep diaries and examined moderation by race/ethnicity.

**Methods:**

Participants from the Study of Women’s Health Across the Nation (SWAN) Sleep Study included 323 White (*n* = 147), African American (*n* = 120), and Chinese (*n* = 56) middle-aged community-dwelling women (mean age: 51 years, range: 48–57). PSG, wrist actigraphy (AW-64; Philips Respironics, McMurray, PA), and sleep diaries were collected concurrently in participants’ homes over three consecutive nights. Multivariable repeated-measures linear models compared time in bed (TIB), total sleep time (TST), sleep efficiency (SE), sleep latency (SL), and wake after sleep onset (WASO) across modalities.

**Results:**

Actigraphy and PSG produced similar estimates of sleep duration and efficiency. Diaries yielded higher estimates of TIB, TST, and SE versus PSG and actigraphy, and lower estimates of SL and WASO versus PSG. Diary SL was shorter than PSG SL only among White women, and diary WASO was lower than PSG and actigraphy WASO among African American versus White women.

**Conclusions:**

Given concordance with PSG, actigraphy may be preferred as an alternative to PSG for measuring sleep in the home. Future research should consider racial/ethnic differences in diary-reported sleep continuity.

**Statement of Significance**

Measuring sleep in the home is more ecologically valid and less burdensome than laboratory-based sleep assessment. However, it is unclear how different sleep measurement modalities perform relative to one another in this natural environment. This in-home study compared indices of sleep duration and continuity between polysomnography (PSG), actigraphy, and diaries across three consecutive nights in a racially/ethnically diverse sample of 323 midlife women. Actigraphy and PSG yielded comparable estimates of most sleep indices, including clinically relevant sleep disturbances, while diary estimates consistently differed from PSG and actigraphy. Modality differences in sleep continuity were not uniform across race/ethnicity. These findings suggest that actigraphy, but not sleep diaries, yield similar results to PSG among midlife women when measured in the home.

## Introduction

Sleep measurement methods may influence the results and interpretation of epidemiological, experimental, and clinical sleep studies, emphasizing the importance of understanding how various sleep assessment modalities compare to one another. In addition to practical and logistical factors, such as cost and participant burden, the selection of measurement modality is often dictated by the outcome of interest. For example, polysomnography (PSG) may be used to quantify physiological characteristics of sleep (e.g. sleep architecture) and nocturnal physiology (e.g. sleep-disordered breathing [SDB], autonomic activity during sleep) [1], whereas self-report may be used to assess qualitative dimensions of sleep (e.g. how rested one feels upon awakening) [2]. Wrist actigraphy, in which sleep is inferred from lack of movement, is useful for measuring naturalistic rest-activity patterns and habitual sleep, as data are collected continuously and noninvasively over many days [3, 4]. While some outcomes are unique to a specific measurement modality, indices of sleep duration and continuity can be measured by multiple modalities including objective (e.g. physiological [PSG] and behavioral [actigraphy]) and subjective (e.g. self-report) assessments. PSG, actigraphy, and self-report sleep diaries are three primary modalities by which sleep is measured.

Because PSG directly measures brain electrophysiology, it is considered the “gold standard” measure for many sleep outcomes against which actigraphy and self-report are compared. Despite its status as the benchmark sleep measure, PSG has several limitations, including high cost (equipment, signal processing, expert personnel) and participant burden [5], even when performed in-home and unattended. These shortcomings are compounded when data are collected across multiple nights, which is desirable due to the potential impact of study procedures on sleep (e.g. “first night effect”) and the natural night-to-night variability in many sleep outcomes [6–9]. Habitual aspects of sleep and variability in sleep patterns both inform our understanding of normative and disordered sleep and their influence on health, functioning, and mortality [10–12], so actigraphy and self-report sleep diaries may be preferred over PSG.

Using participant self-report, daily sleep diaries ascertain habitual sleep characteristics including time in and out of bed, timing of sleep and wake, and the number, duration, and reasons for awakening after sleep onset [13]. However, diaries may suffer from recall bias and incur more participant burden than wrist actigraphy. Actigraphy, while having lower burden and being more objective than diaries, exhibits poor specificity for discriminating wake from sleep when activity is low [14] and may mis-score off-wrist activity as sleep [15]. Actigraphy and sleep diaries have unique clinical utility, as they are recommended for in-home assessment of sleep disorders, including insomnia and circadian rhythm sleep-wake disorders (CRSWDs) [16, 17]. Given the practicality and clinical relevance of actigraphy and sleep diaries, it is necessary to understand how well these modalities compare to PSG in the home setting where they are often used. Furthermore, because the cost of actigraphy (equipment, data processing, and cleaning) may hinder implementation, it is important to consider how diary estimates of sleep compare to actigraphy.

The few studies that have compared PSG, actigraphy, and sleep diaries concurrently have reported that actigraphy yields comparable estimates of sleep duration to PSG [18], but mixed evidence for diary compared to PSG and actigraphy [18, 19]. Actigraphy produces similar estimates as PSG on most other sleep parameters but yields consistently lower sleep latency (SL) estimates compared to PSG [18]. These studies are characterized by various limitations to ecological validity and generalizability, including small sample sizes [18, 19], a single night of assessed sleep [19], study of individuals with sleep or mental health disorders [18, 19], and administration in laboratory settings [18, 19]. A meta-analysis of studies comparing actigraphy and PSG in non-laboratory settings found that actigraphy largely exhibited high agreement with PSG, yet also estimated longer sleep duration and greater sleep continuity than PSG. Agreement between modalities decreased with worsening sleep quality [20]. To our knowledge, only one previous study has compared sleep across all three modalities in the home [21], finding that self-report diaries yielded longer estimates of sleep duration—the only sleep outcome measured—compared to actigraphy and PSG. These results suggest that other sleep outcomes, such as indices of sleep continuity and clinically relevant sleep disturbances, may also differ by measurement modality, but these questions have not been previously tested.

Aging affects sleep, and sleep problems in women are especially prevalent during the late reproductive (perimenopausal) stages and across the menopausal transition, which may be a key inflection point when sleep patterns are altered negatively. Previous studies have shown that subjective sleep complaints persist during peri- and post-menopause [22]. Because there are potential differences between objectively and subjectively measured sleep in women [23, 24], in this analysis we compared sleep duration and continuity measured both subjectively and objectively. Therefore, the present study compared measures of sleep duration (time in bed [TIB], total sleep time [TST]), and continuity (sleep efficiency [SE], SL, wakefulness after sleep onset [WASO]) assessed by PSG, wrist actigraphy, and sleep diaries across up to three nights in a community sample of 323 midlife women. Clinically relevant sleep disturbances (e.g. short sleep duration, difficulties maintaining sleep) were also compared between modalities. All data were collected in participants’ homes over three consecutive nights, which permitted a direct comparison of measures for the same nights across all three modalities. Each of the five sleep indices (TIB, TST, SE, SL, and WASO) can be measured by all three modalities and have been variously related to health, functioning, and mortality [25–31].

Given that associations between diary- and actigraphy-assessed sleep [32] and diary- and PSG-assessed sleep duration [21] have been shown to differ between African American and White adults, race/ethnicity was explored as a potential effect modifier. Several other factors may affect agreement between sleep measurement modalities. Vasomotor symptoms (VMS) have been associated with greater motor restlessness in bed [33], which may affect actigraphy more than diaries and PSG. Individuals who were obese self-reported shorter sleep at similar levels of actigraphy-measured sleep compared to those who were not obese [32]. The use of medications that affect sleep [34] and depressive symptoms [21] have both been associated with greater discrepancy between diary- and actigraphy-measured TST, resulting in shorter diary- versus actigraphy-assessed TST [21, 34]. These factors were examined as covariates in the present analyses.

## Method

### Study participants

The multi-modal Study of Women’s Health Across the Nation (SWAN) Sleep Study was an ancillary study, conducted in a subset of the multi-racial/ethnic cohort of midlife women of SWAN [35]. SWAN is a community-based, longitudinal study of the menopausal transition and its relationships with health and aging, originally enrolling 3302 women. The following exclusion criteria were applied to SWAN participants to determine eligibility for the SWAN Sleep Study: hysterectomy or bilateral oophorectomy (<1% of the cohort), hormone therapy use (23%), nonadherence with core SWAN procedures (missed more than half of annual visits), and biobehavioral factors known to affect sleep, including regular shift/night work, oral corticosteroid use, active treatment for cancer, or alcohol consumption exceeding four drinks per day (1%–3% for each). All eligible participants were approached regarding participation. Of these, 30% declined, with the most cited reasons including “protocol burden,” “too busy,” and “family obligations.” The SWAN Sleep Study enrolled 370 White, African American, and Chinese participants from four of the seven core SWAN study sites: Chicago, IL; Detroit, MI; Oakland, CA; and Pittsburgh, PA.

The present analyses excluded 47 (13%) Sleep Study participants who lacked at least one night of concurrent PSG, actigraphy, and sleep diary data, resulting in an analytic sample of 323. No other inclusion/exclusion criteria were applied. Included participants did not differ from excluded Sleep Study participants on age (*t*[368] = 0.58, *p* = .56), race/ethnicity (χ ^2^[2, *N* = 370] = 3.69, *p* = .16), education (χ ^2^[2, *N* = 364] = −3.98, *p* = .14), body mass index (BMI) (*t*[362] = −0.87, *p* = .38), sleep quality (*t*[364] = 1.23, *p* = .22), or use of medications that affect sleep (χ ^2^[1, *N* = 370] = 0.27, p = 0.60), defined using the following World Health Organization Anatomical Therapeutic Chemical classifications [36]: opioids, antiepileptics, anxiolytics, hypnotics and sedatives, antidepressants, and antihistamines. A smaller proportion of smokers were in the included vs. excluded participants (χ ^2^[1, *N* = 365] = 17.21, *p* < .001). Informed consent was obtained in accordance with approved protocols and guidelines of the Institutional Review Board at each participating institution. Participants were paid for their participation.

### Study protocol

The SWAN Sleep Study protocol [37] was conducted across an entire menstrual cycle or 35 days, whichever was shorter. Unattended PSG sleep studies were conducted in participants’ homes on the first three nights of the protocol. Study staff arrived at participants’ homes approximately 3 h before the participants’ bedtime to apply electrodes and calibrate monitors. Participants slept in their own beds and went to bed and awoke according to their habitual sleep and wake times, which were determined by self-report. Participants turned off the PSG recorder and removed study equipment themselves upon awakening in the morning. Wrist actigraphy and sleep diary data were collected throughout the protocol. Other measures pertinent to the current analyses were collected in conjunction with the Sleep Study or core SWAN protocol, as described below.

### Sleep

Each participant contributed one to three nights of concurrent PSG, wrist actigraphy, and sleep diary data. Sleep outcomes included in the present study were variables that could be measured by all three measurement modalities: indices of sleep duration (TIB, TST) and sleep continuity (SE, SL, WASO).

### PSG

PSG sleep data were collected with Vitaport-3 (Temec; Kerkade, Netherlands) ambulatory recorders. Signals collected on each study night included bilateral central referential electroencephalogram (EEG) channels (C_3_ and C_4_, referenced to A_1_–A_2_), electro-oculogram (EOG), submentalis electromyogram (EMG), and electrocardiogram (EKG). Additional signals were collected on the first night of sleep studies for the assessment of SDB (nasal pressure and oral-nasal thermistors, fingertip oximeter, and abdominal and thoracic excursion, as measured by inductance plethysmography to reflect respiratory effort) and leg movements. Quality assurance assessments, scoring, and processing of all PSG records was performed at the University of Pittsburgh Neuroscience—Clinical and Translational Research Center (N-CTRC) as previously described [37].

Sleep stage scoring was performed by trained PSG technologists with established inter-rater reliability (i.e. intraclass correlation coefficients for wake, non-rapid eye movement, and rapid eye movement each > 0.90) in a sample largely overlapping this study. PSG-assessed. TIB was calculated as time from reported lights out (“got into bed with the intention to go to sleep”) to time of reported awakening from sleep (“awoke in the morning”). Sleep technologists examined PSG records for signs of movement artifact in EEG, EMG, and EOG channels as an indicator of active wakefulness. A persistent reduction in movement artifact across channels was taken as evidence of “settling” that corresponds with lights off and/or attempting to sleep. PSG-assessed TST was calculated as total minutes of any sleep stage after sleep onset. PSG-assessed sleep continuity measures included SL (time from beginning of the recording period to the first of 10 consecutive minutes of Stage 2 or Stage 3–4 sleep interrupted by no more than two minutes of Stage 1 or wakefulness), WASO (total minutes of wakefulness between sleep onset and good morning time [GMT]), and SE (time spent asleep/TIB × 100).

### Actigraphy

Participants wore the Mini-Mitter actiwatch (AW-64; Phillips Respironics, McMurray, PA) on their nondominant wrist throughout the duration of the protocol. This device has been validated against PSG [38]. Data were uploaded for later processing and scoring in 1-minute epochs using Actiware version 5.04 software standard procedures and the medium sensitivity threshold (40 activity counts per epoch). Actigraphy-assessed TIB was defined by study staff as each day’s suspected nocturnal sleep period: the difference between good night time (GNT)—the time at which participants “got into bed with the intention to go to sleep,” and GMT—the time at which participants “awoke in the morning.” Actigraphy GNT and GMT were informed by GNT and GMT reported in sleep diaries. Within TIB, sleep onset was identified as the first epoch of 10 consecutive minutes of sleep, in which less than one epoch was scored as wake. Actigraphy-assessed TST was calculated as the total number of epochs within TIB scored as sleep after sleep onset. Actigraphy-assessed SL and WASO were calculated as the number of epochs from GNT to sleep onset and the total number of epochs scored as “awake” following sleep onset to GMT, respectively. Actigraphy-assessed SE was calculated as TST/TIB × 100.

### Sleep diaries

Each morning upon awakening, participants recorded information about the previous night’s sleep using a sample-specific version of the Pittsburgh Sleep Diary [39]. Diary variables relevant to the current analyses included GNT, GMT, SL (“last night it took me ___ minutes to fall asleep”), and WASO (“last night I spent ___ minutes awake after falling asleep”). Diary-assessed TIB was calculated as the total number of minutes between GNT and GMT, while TST was calculated as TIB minus SL and WASO. SE was calculated as TST/TIB × 100.

### Covariates

Covariates were measures demonstrated in previous SWAN studies to be strongly related to sleep and included race/ethnicity, VMS, BMI, use of medications that affect sleep, and symptoms of depression [37, 40]. Race/ethnicity (non-Hispanic White, African American, or Chinese) was ascertained by self-report. Each morning upon awakening, participants recorded the total number of hot flashes, cold sweats, and night sweats experienced during the previous night. Due to the distributional properties of VMS in this sample, number of symptoms was averaged across PSG nights and dichotomized as “none” or “at least one” reported. BMI was calculated as weight in kilograms/(height in meters)^2^, as measured by study staff. Self-reported symptoms of depression were assessed on the final PSG night using the 16-item Quick Inventory of Depressive Symptomatology (QIDS) [41]. The QIDS was calculated as a continuous variable (Cronbach’s α for reliability = 0.67, 95% CI [0.61 to 0.72]) without the four-item sleep disturbance subscale to reduce collinearity with sleep outcome variables. Use of medications that affect sleep was operationalized as present or absent.

### Statistical analysis

Analyses were performed in SAS version 9.2. Descriptive statistics were used to characterize the study sample and evaluate data distributions. Prior to analyses, non-normally distributed variables (SE, SL, and WASO) were transformed by natural logarithm or square root. Participants could contribute a maximum of three nights of data for each of the three measurement modalities; contributing all nine possible data points was considered complete data. A total of 262 (81%) participants provided complete data, 53 (16%) provided eight data points, 7 (2%) provided seven data points, and 1 (<1%) provided six data points.

A series of multivariable linear regression models with correlated errors over repeated measures, a class of linear mixed effects models, were performed for each of the five sleep variables, adjusting for race/ethnicity, BMI, VMS, symptoms of depression, and medications that affect sleep. Models were fit with maximum likelihood estimation using SAS Proc MIXED. Time within participant and modality within participant were included as random effects and a categorical temporal fixed effect was included to allow sleep measures to vary across the three nights. A first-order autoregressive error structure was used to model the within-participant correlation over time, while an unstructured correlation structure was used to model the correlation of sleep as measured by different modalities for a given participant on a given night.

To allow covariates to interact with different modalities while offering parsimonious models, a step-down model selection procedure was implemented for each sleep variable. This procedure started with an initial model that included all main effects and two-way interactions between covariates, modality, and night. The reference group, used to compare specific values across measurement modalities, was White women of average BMI, low depressive symptoms, no use of medications that affect sleep, and no VMS. Race/ethnicity was the only covariate that interacted significantly with modality and was, therefore, the only covariate retained as an interaction term. Wald tests and confidence intervals were used for performing inference, and residual-based diagnostics were used to assess model fit; *p*-values were not corrected for multiple comparisons.

For each sleep variable, the Bland-Altman approach [42] was used to evaluate whether the observed values assessed by any pair of measurement modalities (e.g. actigraphy and PSG) differed as a function of the size of measurement across modalities. Plots of the mean difference and 95% limits of agreement (LoAs) were generated using recent guidelines [43]. In addition, McNemar’s Test [44] was used to evaluate whether identification of clinically significant sleep disturbances differed as a function of modality. Clinically significant sleep disturbances were defined as follows: TST <6 h, SL >30 min, WASO >30 min, and SE <85% [16, 45]. Long sleep duration (i.e. TST > 9 h) was not considered due to the paucity of long sleepers in our sample (*n* = 3).

## Results

Participants were midlife women between 48 and 57 years of age (mean = 51.2 ± 2.2 years). Self-identified race/ethnicity was: White (*n* = 147), African American (*n* = 120), and Chinese (*n* = 56). Average BMI in the sample was 29.7 (± 7.7), and one quarter of the sample endorsed use of medications that affect sleep (25.7%). Scores for depressive symptoms were low (mean QIDS score = 4.8 ± 3.0; clinical cutoff for QIDS is 13). BMI differed between groups (*F*[2, 309] = 44.47, *p* < .001) such that Chinese women had lower BMI than White and African American women (*p*s < .001) and White women had lower BMI than African American women (*p* < .001). VMS differed between groups (χ ^2^[2, *N* = 317] = 6.39, *p* = .04); presence of VMS by group was: White (29.9%), African American (40.3%), and Chinese (22.2%). Depressive symptoms (*F*[2, 311] = 0.85, *p* = .43) and medication use (χ ^2^[2, *N* = 323] = 1.36, *p* = .51) did not differ between groups.

### Main effects of modality

Descriptive means and mean differences for each sleep outcome across each of the three measurement modalities in the full sample are presented in Table 1. Model fit was acceptable for all models (see residual-based model fit statistics in Supplementary Figure 1A–E). Results from the repeated-measures linear models showed that diary-assessed indices of sleep duration (TIB, TST) and SE were significantly higher than values obtained by PSG and by actigraphy. On average, diary-assessed TIB for the reference group was 20.4 (± 3.4) and 18.1 (± 2.3) minutes longer than PSG- and actigraphy-assessed values, respectively. Similarly, diary-assessed TST for the reference group was 12.6 (± 4.9) and 21.2 (± 4.9) minutes longer on average than values derived from PSG and actigraphy, respectively. Diary-assessed SE was 7.2% (± 1.1) and 7.0% (+/- 1.1) higher on average than PSG- and actigraphy-assessed values, respectively. Actigraphy-assessed indices of sleep duration (TIB, TST) and SE did not significantly differ from those assessed by PSG (*p*s > .05).

**Table 1.** Sleep outcome means and mean differences by modality in the full sample (*N* = 323)

	Mean (*SD*)			Mean difference (*SD*)		
Sleep measure	PSG	Actigraphy	Diary	PSG-ACT	PSG-Diary	ACT-Diary
Time in bed (min)	451.0 (58.5)	452.0 (71.8)	471.4 (69.5)	−0.7 (65.6)	−20.1 (57.6)	−19.5 (46.4)
Total sleep time (min)	374.7 (54.5)	365.5 (67.1)	388.4 (63.0)	9.6 (60.8)	−13.7 (52.1)	−23.0 (60.9)
Sleep latency (min)	22.6 (20.4)	20.7 (37.3)	21.1 (19.8)	2.4 (39.5)	1.4 (23.4)	−0.5 (38.4)
Wake after sleep onset (min)	54.6 (32.4)	45.3 (26.6)	17.4 (22.2)	9.7 (31.7)	37.6 (32.9)	27.8 (30.5)
Sleep efficiency (%)	82.2 (8.1)	80.3 (11.4)	90.0 (7.4)	1.7 (11.9)	−7.8 (8.9)	−9.7 (12.5)

PSG, polysomnography; ACT, actigraphy; *SD*, standard deviation.

Different patterns were observed across modalities for SL and WASO. For SL, PSG values were significantly higher than those obtained by sleep diary (*t*[602] = 5.30, *p* < .001) and by actigraphy (*t*[602] = 9.69, *p* < .001). On average, PSG-assessed SL in the reference group was 8.5 (± 1.4) and 4.0 (± 1.1) minutes longer than actigraphy- and diary-assessed SL, respectively. In turn, diary-assessed SL in the reference group was an average of 4.4 (± 1.1) minutes longer than actigraphy assessment (*t*[602] = 5.15, *p* < .001). WASO was higher when measured by PSG compared to both actigraphy (*t*[602] = 18.12) and sleep diary (*t*[602] = 3.51) (*p*s < .001), while actigraphy-assessed WASO was 21.9 (± 3.0) minutes longer than that reported by sleep diaries (*t*[602] = 8.54, *p* < .001). PSG-assessed WASO in the reference group was an average of 7.5 (± 2.1) and 29.6 (± 3.4) minutes longer than values derived from actigraphy and sleep diaries, respectively.

### Interactions of race and night by modality

We next examined whether modality differences for indices of sleep duration and continuity differed as a function of race/ethnicity or night of study (Table 2). Significant race/ethnicity-by-modality interactions were observed for SL (*F*[4, 2301] = 3.15, *p* = .014) and WASO (*F*[4, 2305] = 5.56, *p* < .001). Post-hoc contrasts revealed a significant difference between diary- and PSG-assessed SL in White participants, which was larger than in African American (*F*[1, 2301] = 4.05, *p* = 0.04) and Chinese women (*F*[1, 2301] = 10.51, *p* = .001). In contrast, the difference between diary- and PSG-assessed WASO was significantly larger in African American participants compared to Whites (*F*[1, 2305] = 20.16, *p* < .001) and tended to be larger than the difference observed in Chinese participants (*F*[1, 2305] = 5.93, *p* = .015). The difference between diary- and actigraphy-assessed WASO was also significantly larger in African American compared to White participants (*F*[1, 2305] = 11.94, *p* < .001) but was similar to Chinese participants (*F*[1, 2305] = 2.24, *p* = .13). Race-by-modality interactions were not observed for indices of sleep duration (TIB, TST) or SE. None of the modality-by-night interactions was significant, suggesting that modality effects were consistent across recording nights.

**Table 2.** Estimated sleep outcomes by modality and race/ethnicity from fully adjusted models (*N* = 323)

Sleep measure	Mean (*SE*)			Mean difference (*SE*)		
	PSG	Actigraphy	Diary	PSG-ACT	PSG-Diary	ACT-Diary
Time in bed (min)						
White	430.7 (7.8)	428.1 (8.1)	442.4 (8.1)	2.5 (5.6)	−11.7 (5.2)*	−14.2 (3.6)***
African American	417.6 (8.6)	431.0 (8.9)	448.4 (8.9)	−13.4 (6.8)*	−30.8 (5.7)***	−17.5 (4.3)***
Chinese	415.4 (10.9)	412.1 (11.5)	432.3 (11.4)	3.3 (8.8)	−16.9 (8.1)*	−20.2 (5.6)***
Total sleep time (min)						
White	373.9 (6.6)	365.3 (6.9)	386.5 (7.2)	8.6 (4.9)	−12.6 (4.9)*	−21.2 (5.0)***
African American	340.6 (7.3)	341.0 (7.6)	366.9 (8.0)	−0.4 (6.0)	−26.3 (5.9)***	−25.9 (6.0)***
Chinese	363.4 (9.2)	359.5 (7.2)	373.4 (10.2)	3.9 (7.8)	−10.0 (7.7)	−13.9 (7.8)
Sleep latency (min)						
White	11.9 (0.9)	3.4 (0.5)	7.9 (0.7)	8.5 (1.4)***	4.0 (1.1)***	−4.4 (1.1)***
African American	14.6 (1.2)	5.4 (0.9)	12.2 (1.2)	9.2(1.4)***	2.3 (1.2)	−6.9 (1.1)***
Chinese	9.7 (1.1)	2.8 (0.7)	10.2 (1.3)	6.9 (1.6)***	−0.5 (1.2)	−7.4 (1.1)***
Wake after sleep onset (min)						
White	38.6 (2.5)	31.3 (2.2)	9.3 (1.3)	7.5 (2.1)***	29.6 (3.4)***	21.9 (3.0)***
African American	45.1 (3.0)	35.2 (2.6)	5.7 (1.1)	10.3 (2.0)***	39.8 (5.8)***	29.5 (5.1)***
Chinese	36.9 (3.5)	30.6 (3.2)	6.6 (1.5)	6.3 (3.3)	30.4 (6.0)***	24.1 (1.9)***
Sleep efficiency (%)						
White	87.8 (0.6)	88.0 (0.7)	95.0 (0.4)	−0.2 (0.6)	−7.2 (1.1)***	−7.0 (1.1)***
African American	84.8 (0.8)	83.8 (1.0)	94.3 (0.5)	1.0 (1.0)	−9.5 (1.2)***	−10.5 (1.2)***
Chinese	88.6 (0.8)	88.7 (0.9)	94.9 (0.6)	−0.0 (0.9)	−6.3 (1.2)***	−6.2 (1.2)***

Covariates included vasomotor symptoms, BMI, symptoms of depression, and use of medications that affect sleep.

PSG, polysomnography; ACT, actigraphy; *SE*, standard error.

*p < .05.

^***^ *p* < .001.

### Modality effects across the spectrum of measurement

**Figure 1.**
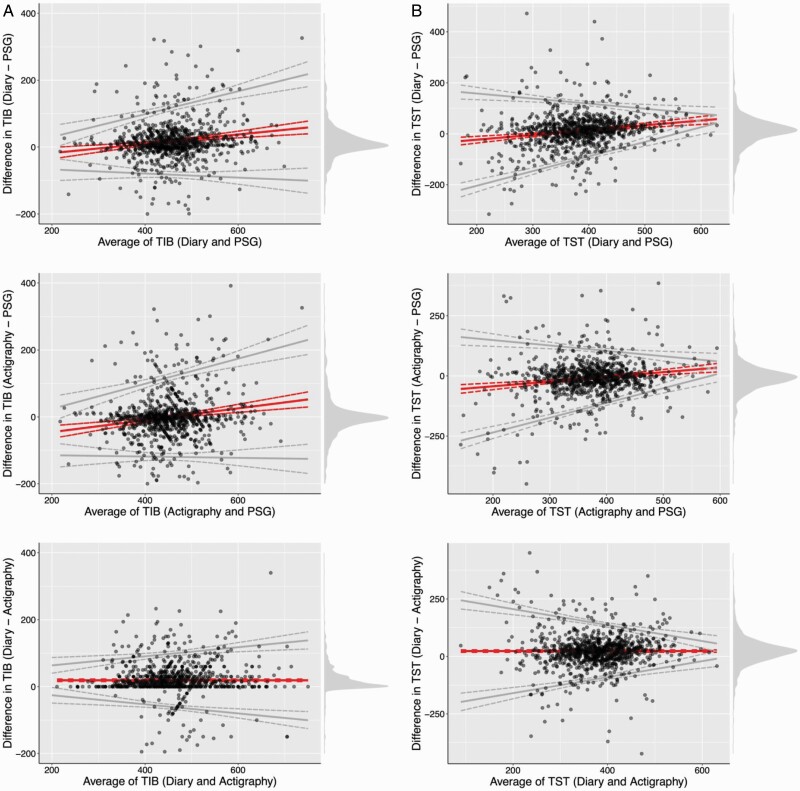

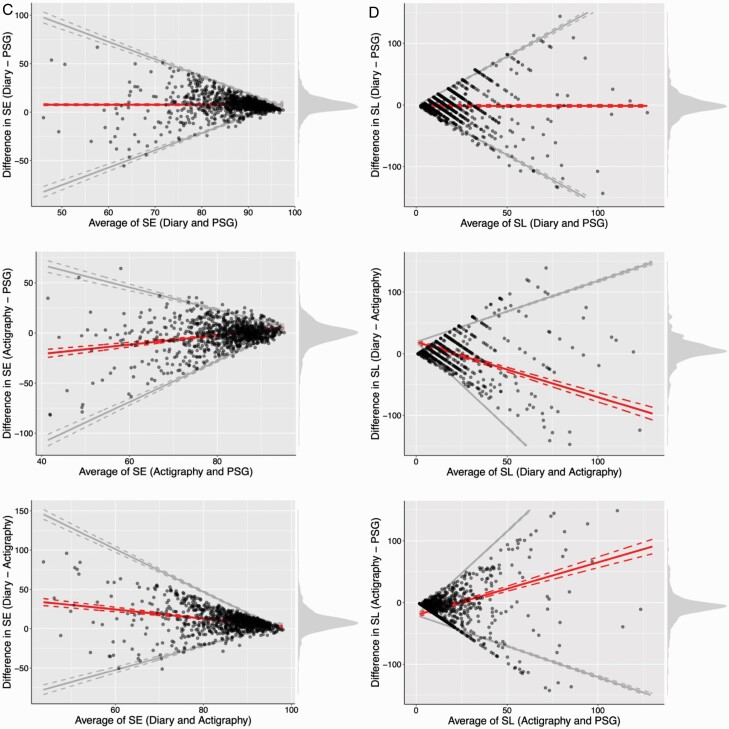

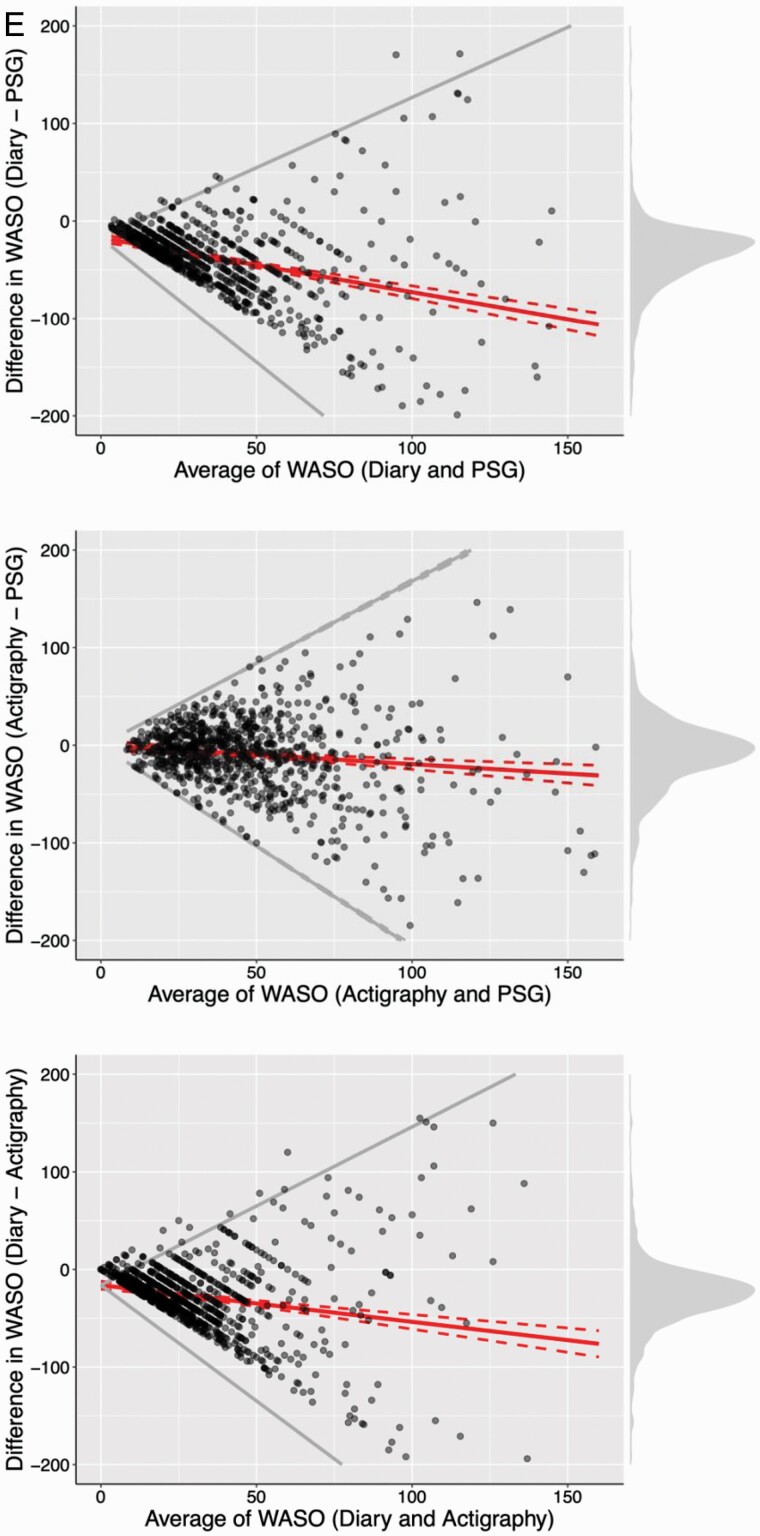
(A–E) Bland-Altman plots comparing indices of sleep duration and continuity between measurement modalities. The red line indicates the observed mean difference (bias) between measurement modalities, while the gray lines reflect 95% limits of agreement (LoAs). All lines are plotted with their 95% confidence intervals (dashed lines). Gray dots are individual nightly observations, and darker shading indicates overlapping observations. The density distribution of observed differences is plotted on the right. Due to high heteroscedasticity, wakefulness after sleep onset and sleep latency were log-transformed to calculate LOAs and back-transformed for plotting purposes. PSG, polysomnography.

Bland-Altman plots were used to evaluate potential biases and LoAs between all three modalities (i.e. diary vs. PSG, actigraphy vs. PSG, and diary vs. actigraphy) for each sleep outcome (Figure 1A–E). A mean difference near zero indicates no systematic bias between two modalities. Systematic biases depicted in the figures are consistent with results of mixed model analyses. The slope of the mean difference indicated that diaries yielded higher estimates of TIB, TST, and WASO versus PSG as the size of measurement increased. Mean difference slopes also showed that as the size of measurement increased, actigraphy produced higher estimates of all five sleep outcomes versus PSG and diaries yielded lower SE, SL, and WASO estimates versus actigraphy. Heteroscedasticity, representing increasing or decreasing variability with size of measurement, is indicated by 95% LoAs. Heteroscedasticity was observed for all sleep outcomes and modalities: variability increased with longer TIB and shorter TST and increased substantially with poorer values of sleep continuity (i.e. lower SE, higher SL, and WASO).

### Clinically relevant sleep disturbances by modality

Compared to PSG, sleep diaries indicated a lower prevalence of short sleep duration (TST < 6 h; χ ^2^[1, *N* = 323] = 12.13) and difficulties maintaining sleep (WASO > 30 min, χ ^2^[1, *N* = 323] = 42.38; SE < 85%, χ ^2^[1, *N* = 323] = 60.38) (*p*s < .001) (Table 3). Diaries also demonstrated a non-significant trend toward lower prevalence estimates of difficulties initiating sleep (SL >30 min; χ ^2^[1, *N* = 323] = 3.52, *p* = .06) versus PSG, while actigraphy yielded a significantly higher prevalence of difficulty initiating sleep (χ ^2^[1, *N* = 323] = 4.90, *p* < .03) versus PSG. For short sleep duration and difficulty maintaining sleep, approximately one-quarter of participants were differentially classified across each modality comparison. Larger differences were observed for difficulties maintaining sleep, especially for PSG versus diary and actigraphy versus diary (48%–67% changed categories). These findings are supported by the exceptionally wide variability between modalities in the Bland-Altman plots.

**Table 3.** Clinically significant sleep disturbances by measurement modality (*N* = 323)

Sleep measure	*N* in each category (%)			*N* changed categories (%)		
	PSG	ACT	Diary	PSG-ACT	PSG-Diary	ACT-Diary
Total sleep time	110 (34%)	129 (40%)	93 (29%)	77 (24%)	73 (23%)	82 (25%)
< 360 min						
Sleep latency	64 (20%)	53 (16%)	66(20%)	79 (25%)	79 (25%)	77 (25%)
> 30 min						
Wake after sleep onset	259 (80%)	224 (70%)	53 (16%)	103 (32%)	215 (67%)	197 (61%)
> 30 min						
Sleep efficiency	177 (55%)	186 (58%)	55 (17%)	136 (42%)	153 (48%)	175 (54%)
< 85%						

*N* in each category (%) refers to the number and percentage, respectively, of participants meeting a given threshold for clinically significant sleep disturbance when measured by each modality. *N* changed categories (%) refers to the number and percentage, respectively, of participants who met the given threshold when measured by one given modality but not the other. For example, in the “PSG-ACT” column, a participant with 350 min of PSG-assessed TST and 370 min of actigraphy-assessed TST would change categories, as would a participant with 370 min of PSG-assessed TST and 350 min of actigraphy-assessed TST.

PSG, polysomnography; ACT, actigraphy; TST, total sleep time.

## Discussion

To our knowledge, this is the largest study to date to directly compare indices of sleep duration and continuity assessed concurrently by PSG, wrist actigraphy, and sleep diaries. We found that mean estimates of sleep duration and SE were similar in actigraphy and PSG but higher in sleep diaries. Both diaries and actigraphy yielded lower estimates of SL and WASO compared to PSG, although differences in diary-assessed SL and WASO varied by race/ethnicity. All modalities showed less agreement with each other at values of poorer sleep: longer TIB, shorter TST, lower SE, and higher SL and WASO. Compared to PSG, sleep diaries identified a lower prevalence of clinically meaningful short sleep and poor sleep continuity, while diary and actigraphy estimated lower and higher prevalence of SL, respectively. These findings suggest that actigraphy measures many important sleep parameters comparably to in-home PSG, but diaries consistently differ from both PSG and actigraphy.

Actigraphy and PSG produced similar estimates of TIB and TST, but diaries yielded longer estimates of sleep duration compared to both actigraphy and PSG. These results are mostly in line with previous studies comparing TST and TIB across modalities [21, 46–52], with the few exceptions being in patients with insomnia in which diary-assessed TST was longer than TST measured by actigraphy [53] and PSG [19]. Our results show that actigraphy and PSG perform comparably on indices of sleep duration in the home, but diaries estimate longer sleep duration than actigraphy and PSG.

Estimates of SE were also comparable between actigraphy and PSG, while diaries measured higher SE relative to both modalities. This actigraphy-PSG agreement is consistent with past research [46–49], as are the higher SE values estimated by diaries versus actigraphy [50]. Differences between diaries and actigraphy were likely explained by diaries yielding increasingly higher SE values than actigraphy at lower SE. Although the mean SE difference between actigraphy and PSG in our study was small, variability between measures increased as SE decreased, consistent with prior studies [46, 49, 54]. Our findings suggest that modalities may not be reliably comparable in individuals with poor SE (e.g. insomnia).

Modality differences between other indices of sleep continuity (SL and WASO) were complex. Diaries estimated lower SL and WASO values compared to PSG, opposite of the findings of another study, which reported that diaries estimated higher SL and WASO versus PSG in individuals with clinical depression and insomnia [19]. However, as higher subjective vs. objective sleep complaints are a defining feature of insomnia [55], our findings are not necessarily in conflict with previous research, given that our participants were not a clinical sample. The disrupted sleep onset process interferes with this process. Differences between the present study and previous findings may also be related to poorer correspondence among measurement modalities in individuals with poorer sleep continuity, which is observed in individuals with clinical depression and insomnia [19]. Our finding of actigraphy estimating lower SL values relative to PSG is consistent with a recent systematic review that determined that actigraphy generally yields SL estimates up to 10 minutes shorter than PSG, although differences were not often statistically significant, due in part to high inter-individual variability between modalities [56]. Our data suggest that midlife women self-report significantly shorter times falling asleep and waking during sleep relative to actigraphy and PSG. However, it should be noted that sleep onset is associated with a small amount of retrograde amnesia [57], which limits the amount of recalled time spent falling asleep and may contribute to lower reported SL and WASO compared to actigraphy and PSG. The sleep onset process is compromised in insomnia [57], which may explain differences between present study findings and those in individuals with insomnia [19].

Observed differences in sleep continuity were not uniform across race/ethnicities. Racial/ethnic differences in sleep are well documented [37, 58, 59], but few studies have examined racial/ethnic differences across sleep measurement modalities. Previous research indicates that actigraphy- and diary-assessed sleep duration correlate less strongly among African American compared to White adults [21, 32]. Similarly, in a nationwide sample of adults, African Americans were less likely to report problems falling asleep than Whites despite being more likely to report SL greater than 30 min [60]. These differences may reflect racial/ethnic differences in beliefs about sleep (e.g. the role of sleep in health and functioning), such as were observed in a qualitative study of African American and White older women [61]. Given both the known racial/ethnic group differences in sleep [38, 58, 59] and the importance of sleep to health and functioning [26–31], more research is needed to understand the impact of measurement modality on sleep in diverse groups, including the impact of measurement modality on replication across race/ethnicity.

In addition to race/ethnicity and included covariates, other factors may have influenced agreement between measurement modalities. Self-reported sleep duration has been more strongly correlated with wrist actigraphy among individuals with a college degree than those without a college degree [32], suggesting that agreement could differ by participants’ educational attainment. However, education was not associated with sleep in the SWAN sample [37]. Movements by a bed partner may alter the inactivity inferred as sleep by actigraphy. Walters *et al*. [62] observed similar diary- and actigraphy-assessed SL but much higher actigraphy-assessed WASO compared to sleep diary among individuals with bed partners, possibly reflecting a scenario in which awakenings were sufficiently short that participants did not remember the next day. Finally, noise from road traffic has been associated with more reported awakenings and worse sleep quality, and effects on sleep were observed by actigraphy [63]. In summary, education, presence/absence of a bedpartner, and noise/neighborhood environment should be considered as potential moderators of modality agreement in future studies.

Our results also highlight inconsistencies across sleep measurement modalities in identifying clinically relevant sleep disturbances. Multiple measurement modalities are often used in conjunction with one another to improve identification and diagnosis of sleep disorders. For example, while self-report is largely recommended to evaluate insomnia and CRSWDs, actigraphy is also used to both characterize sleep disturbances in these conditions and, in the case of CRSWDs, assess response to treatment [16, 17]. Our results indicate that diaries and actigraphy may classify short sleep duration and difficulty falling asleep similarly, but these modalities yield conflicting classifications of poor sleep continuity. Furthermore, differences will likely be exacerbated among individuals with poor sleep continuity. Although classification of clinically relevant sleep disturbances differed widely across measurement modalities, it must be noted that each modality characterizes unique aspects of sleep and may therefore provide clinically valid information depending on the outcome of interest.

Although midlife women generally report a high prevalence of sleep complaints [64–66], particularly in the context of physiological changes associated with the menopausal transition [67–69], our findings suggest that self-reported sleep was endorsed as more favorable (i.e. shorter TST, lower WASO, higher SE) compared to actigraphy and PSG. Furthermore, Bland-Altman plots indicated that differences between subjectively and objectively measured sleep continuity may be significantly greater in midlife women with more sleep disturbances, which is consistent with a model [57] in which individuals with good sleep underestimate SL and WASO, while individuals with insomnia overestimate relative to these objective measures. Our results highlight the need for better assessments of sleep disturbances in midlife women.

Several limitations and strengths should be considered when evaluating the present results and their implications. Although our study is unique in measuring sleep with objective (i.e. physiological [PSG] and behavioral [actigraphy]) and subjective (i.e. diaries) modalities in the home across three nights in a large and diverse sample of midlife women, results may not be generalizable to other populations. Characteristics of the menopausal transition, including its known effects on nocturnal physiology, may limit the degree to which these findings can be extended to women at other points in developmental or reproductive stages. In addition, results cannot be generalized to men, other age groups, or other racial/ethnic groups. More research should evaluate the impact of measurement modality on sleep given known changes in sleep across the lifespan [70] and differences in sleep by sex [71] and across racial/ethnic groups [72]. Finally, the exclusive use of the AW-64 medium sensitivity threshold limits generalization of findings to other sensitivity thresholds for this device. Low and high sensitivity thresholds can better detect wakefulness and sleep, respectively [73], so using alternate thresholds may have impacted the magnitude, but not the overall pattern, of observed modality differences. Despite these limitations, the present study has numerous strengths, including a rigorous design, a large and racially/ethnically diverse sample, consideration of numerous potential covariates, data collection using standardized protocols across all clinical sites, and high ecological validity via in-home assessment where participants adhered to their natural sleep-wake schedules.

In summary, we found that self-report sleep diaries yielded longer estimates of sleep duration and more favorable estimates of sleep continuity (i.e. lower WASO and higher SE) in comparison to objectively assessed actigraphy and PSG in midlife women. Differences were seen across up to three nights of sleep and, overall, were similar for White, African American, and Chinese women. Actigraphy and PSG produced similar estimates of sleep duration and efficiency. Our findings suggest that actigraphy may be recommended as a lower-cost alternative to PSG to assess sleep among midlife women in home settings. Observed differences between diaries and actigraphy and PSG should be considered when interpreting results from actigraphy and in-home PSG in the context of sleep diaries, specifically because self-report diaries are likely to estimate longer sleep duration and greater sleep continuity than these objective modalities. However, we emphasize that each modality captures unique aspects of sleep, and modality differences should not be interpreted as measurement error per se. Results of the present study may not be generalizable to patients with clinical sleep disorders, such as insomnia or sleep apnea, in which large differences between self-report and actigraphy- or PSG-assessed sleep are common, or to men or to other age groups. Continued efforts to better understand differences in sleep outcomes vis-à-vis measurement modality, and factors that influence these differences, remain critical to our understanding of sleep, the diagnosis and treatment of sleep disorders, and the importance of sleep to health and functioning.

## Supplementary Material

zpac001_suppl_Supplementary_Material

## Data Availability

The data underlying this article are from an ongoing multisite study and are not available to be shared.

## References

[1] Kushida CA, et al Practice parameters for the indications for polysomnography and related procedures: an update for 2005. Sleep. 2005;28(4):499–521. doi:10.1093/sleep/28.4.499. 16171294

[2] Ibáñez V, et al A survey on sleep assessment methods. PeerJ. 2018;25(6):e4849. 10.7717/peerj.4849 PMC5971842 29844990

[3] Ancoli-Israel S, et al The role of actigraphy in the study of sleep and circadian rhythms. Sleep. 2003;26(3):342–392. doi:10.1093/sleep/26.3.342. 12749557

[4] de Souza L, et al Further validation of actigraphy for sleep studies. Sleep. 2003;26(1):81–85. doi:10.1093/sleep/26.1.81. 12627737

[5] Walia HK, et al Practical aspects of actigraphy and approaches in clinical and research domains. In: Levin KH, Chavel P, eds. Handbook of Clinical Neurology. Vol. 160. Amsterdam, Netherlands: Elsevier; 2019:371–379. 31277861 10.1016/B978-0-444-64032-1.00024-2

[6] Newell J, et al Is a one-night stay in the lab really enough to conclude? First-night effect and night-to-night variability in polysomnographic recordings among different clinical population samples. Psychiatry Res. 2012;200(2–3):795–801. 22901399 10.1016/j.psychres.2012.07.045

[7] Israel B, et al Short-term stability of sleep and heart rate variability in good sleepers and patients with insomnia: for some measures, one night is enough. Sleep. 2012;35(9):1285–1291. doi:10.5665/sleep.2088. 22942507 PMC3413806

[8] Zheng H, et al Sources of variability in epidemiological studies of sleep using repeated nights of in-home polysomnography: SWAN Sleep Study. J Clin Sleep Med. 2012;8(1):87–96. 22334814 10.5664/jcsm.1670 PMC3266336

[9] Byun JH, et al The first night effect during polysomnography, and patients’ estimates of sleep quality. Psychiatry Res. 2019;274:27–29. 30776709 10.1016/j.psychres.2019.02.011

[10] Buysse DJ . Sleep health: can we define it? Does it matter? Sleep. 2014;37(1):9–17. doi:10.5665/sleep.3298. 24470692 PMC3902880

[11] Bei B, et al Beyond the mean: a systematic review on the correlates of daily intraindividual variability of sleep/wake patterns. Sleep Med Rev. 2016;28:108–124. 26588182 10.1016/j.smrv.2015.06.003

[12] Aurora RN, et al Habitual sleep duration and all-cause mortality in a general community sample. Sleep. 2016;39(11):1903–1909. doi:10.5665/sleep.6212. 27450684 PMC5070744

[13] Carney CE, et al The consensus sleep diary: standardizing prospective sleep self-monitoring. Sleep. 2012;35(2):287–302. doi:10.5665/sleep.1642. 22294820 PMC3250369

[14] Marino M, et al Measuring sleep: accuracy, sensitivity, and specificity of wrist actigraphy compared to polysomnography. Sleep. 2013;36(11):1747–1755. doi:10.5665/sleep.3142. 24179309 PMC3792393

[15] Grandner MA, et al Actigraphic sleep tracking and wearables: historical context, scientific applications and guidelines, limitations, and considerations for commercial sleep devices. In: Sleep and Health; 2019:147–157.

[16] Schutte-Rodin S, et al Clinical guideline for the evaluation and management of chronic insomnia in adults. J Clin Sleep Med. 2008;4(5):487–504. 18853708 PMC2576317

[17] Morgenthaler TI, et al Practice parameters for the clinical evaluation and treatment of circadian rhythm sleep disorders. Sleep. 2007;30(11):1445–1459. doi:10.1093/sleep/30.11.1445. 18041479 PMC2082098

[18] Kaplan KA, et al Evaluating sleep in bipolar disorder: comparison between actigraphy, polysomnography, and sleep diary. Bipolar Disord. 2012;14(8):870–879. 23167935 10.1111/bdi.12021 PMC3549461

[19] McCall C, et al Comparison of actigraphy with polysomnography and sleep logs in depressed insomniacs. J Sleep Res. 2012;21(1):122–127. 21447050 10.1111/j.1365-2869.2011.00917.x PMC3134551

[20] Van De Water AT, et al Objective measurements of sleep for non-laboratory settings as alternatives to polysomnography—a systematic review. J Sleep Res. 2011;20:183–200. 20374444 10.1111/j.1365-2869.2009.00814.x

[21] Matthews KA, et al Similarities and differences in estimates of sleep duration by polysomnography, actigraphy, diary, and self-reported habitual sleep in a community sample. Sleep Health. 2018;4(1):96–103. 29332687 10.1016/j.sleh.2017.10.011 PMC5771411

[22] Kravitz HM, et al Sleep trajectories before and after the final menstrual period in the Study of Women’s Health Across the Nation (SWAN). Curr Sleep Med Rep. 2017;3(3):235–250. 28944165 10.1007/s40675-017-0084-1 PMC5604858

[23] van den Berg JF, et al Sex differences in subjective and actigraphic sleep measures: a population-based study of elderly persons. Sleep. 2009;32(10):1367–1375. doi:10.1093/sleep/32.10.1367. 19848365 PMC2753814

[24] Unruh ML, et al Subjective and objective sleep quality and aging in the sleep heart health study. J Am Geriatr Soc. 2008;56(7):1218–1227. 18482295 10.1111/j.1532-5415.2008.01755.x

[25] Ayas NT, et al A prospective study of self-reported sleep duration and incident diabetes in women. Diabetes Care. 2003;26(2):380–384. 12547866 10.2337/diacare.26.2.380

[26] Ayas NT, et al A prospective study of sleep duration and coronary heart disease in women. Arch Intern Med. 2003;163(2):205–209. 12546611 10.1001/archinte.163.2.205

[27] Patel SR, et al A prospective study of sleep duration and mortality risk in women. Sleep. 2004;27(3):440–444. doi:10.1093/sleep/27.3.440. 15164896

[28] Cappuccio FP, et al Quantity and quality of sleep and incidence of type 2 diabetes: a systematic review and meta-analysis. Diabetes Care. 2010;33(2):414–420. 19910503 10.2337/dc09-1124 PMC2809295

[29] Hayashino Y, et al Relation between sleep quality and quantity, quality of life, and risk of developing diabetes in healthy workers in Japan: The High-risk and Population Strategy for Occupational Health Promotion (HIPOP-OHP) Study. BMC Public Health. 2007;7:129. 17597542 10.1186/1471-2458-7-129 PMC1924854

[30] Ensrud KE, et al Sleep disturbances and risk of frailty and mortality in older men. Sleep Med. 2012;13(10):1217–1225. 22705247 10.1016/j.sleep.2012.04.010 PMC3449012

[31] Anothaisintawee T, et al Sleep disturbances compared to traditional risk factors for diabetes development: systematic review and meta-analysis. Sleep Med Rev. 2016;30:11–24. 26687279 10.1016/j.smrv.2015.10.002

[32] Lauderdale DS, et al Self-reported and measured sleep duration: how similar are they? Epidemiology. 2008;19(6):838–845. 18854708 10.1097/EDE.0b013e318187a7b0 PMC2785092

[33] Kravitz HM, et al An actigraphy study of sleep and pain in midlife women—the SWAN Sleep Study. Menopause. 2015;22(7):710–718. 25706182 10.1097/GME.0000000000000379 PMC4481159

[34] Van Den Berg JF, et al Disagreement between subjective and actigraphic measures of sleep duration in a population-based study of elderly persons. J Sleep Res. 2008;17(3):295–302. 18321246 10.1111/j.1365-2869.2008.00638.x

[35] Sowers MF, et al. SWAN: A multi-center, multi-ethnic, community-based cohort study of women and the menopausal transition. In: Lobo R, Marcus R, and Kelsey J, eds. Menopause. New York: Academic Press; 2000.

[36] World Health Organization. Guidelines for ATC classification. http://www.whocc.no/atcddd. Accessed October 27, 2020.

[37] Hall M, et al Race and financial strain are independent correlates of sleep in mid-life women: The SWAN Sleep Study. Sleep. 2009;32(1):73–82. doi:10.5665/sleep/32.1.73. 19189781 PMC2625326

[38] Schade MM, et al Sleep validity of a non-contact bedside movement and respiration-sensing device. J Clin Sleep Med. 2019;15(7):1051–1061. 31383243 10.5664/jcsm.7892 PMC6622509

[39] Monk TH, et al. The Pittsburgh sleep diary. J Sleep Res. 1994;3(2):111–120. 10607115

[40] Campbell IG, et al Evaluation of the association of menopausal status with delta and beta EEG activity during sleep. Sleep. 2011;34(11):1561–1568. doi:10.5665/sleep.1398. 22043127 PMC3198211

[41] Rush AJ, et al The inventory of depressive symptomatology (IDS): psychometric properties. Psychol Med. 1996;26(3):477–486. 8733206 10.1017/s0033291700035558

[42] Bland JM, et al Statistical methods for assessing agreement between two methods of clinical measurement. Lancet. 1986;327(8476):307–310. 2868172

[43] Menghini L, et al A standardized framework for testing the performance of sleep-tracking technology: step-by-step guidelines and open-source code. Sleep. 2021;44(2). doi:10.1093/sleep/zsaa170. PMC7879416 32882005

[44] McNemar Q . Note on the sampling error of the difference between correlated proportions or percentages. Psychometrika 1947;12(2):153–157. 20254758 10.1007/BF02295996

[45] Consensus Conference P, et al Joint consensus statement of the American Academy of Sleep Medicine and Sleep Research Society on the recommended amount of sleep for a healthy adult: methodology and discussion. Sleep. 2015;38(8):1161–1183. doi:10.5665/sleep.4886. 26194576 PMC4507722

[46] Zinkhan M, et al Agreement of different methods for assessing sleep characteristics: a comparison of two actigraphs, wrist and hip placement, and self-report with polysomnography. Sleep Med. 2014;15(9):1107–1114. 25018025 10.1016/j.sleep.2014.04.015

[47] Quante M, et al Actigraphy-based sleep estimation in adolescents and adults: a comparison with polysomnography using two scoring algorithms. Nat Sci Sleep. 2018;10:13–20. 29403321 10.2147/NSS.S151085 PMC5779275

[48] Full KM, et al Validation of a physical activity accelerometer device worn on the hip and wrist against polysomnography. Sleep Health. 2018;4(2):209–216. 29555136 10.1016/j.sleh.2017.12.007 PMC5863644

[49] Mantua J, et al Reliability of sleep measures from four personal health monitoring devices compared to research-based actigraphy and polysomnography. Sensors. 2016;16(5):646. 27164110 10.3390/s16050646 PMC4883337

[50] Campanini MZ, et al Agreement between sleep diary and actigraphy in a highly educated Brazilian population. Sleep Med. 2017;35:27–34. 28619179 10.1016/j.sleep.2017.04.004

[51] Moore CM, et al Actigraphy and sleep diary measurements in breast cancer survivors: discrepancy in selected sleep parameters. Behav Sleep Med. 2015;13(6):472–490. 25117292 10.1080/15402002.2014.940108 PMC4326642

[52] Tomita S, et al Comparison of sleep diary and actigraphy to evaluate total sleep time in hypersomnia patients. Sleep Biol Rhythms. 2013;11(2):65–73.

[53] Nakase-Richardson R, et al Actigraphic and sleep diary measures in veterans with traumatic brain injury: discrepancy in selected sleep parameters. J Head Trauma Rehab. 2016;31(12):136–146. 10.1097/HTR.0000000000000225 26959667

[54] Baandrup L, et al A validation of wrist actigraphy against polysomnography in patients with schizophrenia or bipolar disorder. Neuropsych Dis Treat. 2015;11:2271–2277. 10.2147/NDT.S88236 PMC4559245 26357475

[55] Bianchi MT, et al The subjective–objective mismatch in sleep perception among those with insomnia and sleep apnea. J Sleep Res. 2013;22(5):557–568. 23521019 10.1111/jsr.12046

[56] Scott H, et al A systematic review of the accuracy of sleep wearable devices for estimating sleep onset. Sleep Med Rev. 2020;49:101227. 31901524 10.1016/j.smrv.2019.101227

[57] Perlis ML, et al The mesograde amnesia of sleep may be attenuated in subjects with primary insomnia. Physiol Behav. 2001;74(1–2):71–76. 11564454 10.1016/s0031-9384(01)00545-5

[58] Fuller-Rowell TE, et al Racial disparities in sleep: the role of neighborhood disadvantage. Sleep Med. 2016;27–28:1–8. 10.1016/j.sleep.2016.10.008 PMC5171231 27938909

[59] Carnethon MR, et al Disparities in sleep characteristics by race/ethnicity in a population-based sample: Chicago Area Sleep Study. Sleep Med. 2016;18:50–55. 26459680 10.1016/j.sleep.2015.07.005 PMC4728038

[60] Grandner MA, et al Sleep symptoms, race/ethnicity, and socioeconomic position. J Clin Sleep Med. 2013;9(9):897–905. 23997702 10.5664/jcsm.2990 PMC3746717

[61] Grandner MA, et al Sleep-related behaviors and beliefs associated with race/ethnicity in women. J Natl Med Assoc. 2013;105(1):4–16. 23862291 10.1016/s0027-9684(15)30080-8 PMC3759527

[62] Walters EM, et al Vulnerability and resistance to sleep disruption by a partner: A study of bed-sharing couples. Sleep Health. 2020;6(4):506–512. 32331861 10.1016/j.sleh.2019.12.005

[63] Öhrström E, et al Sleep disturbances from road traffic and ventilation noise—laboratory and field experiments. J Sound Vibrat. 2004;271(1–2):279–296.

[64] Zhang B, et al Sex differences in insomnia: a meta-analysis. Sleep. 2006;29(1):85–93. doi:10.1093/sleep/29.1.85. 16453985

[65] Ohayon MM . Nocturnal awakenings and comorbid disorders in the American general population. J Psychiatr Res. 2008;43(1):48–54. 18374943 10.1016/j.jpsychires.2008.02.001

[66] Owens JF, et al Sleep disturbance in healthy middle-aged women. Maturitas. 1998;30(1):41–50. 9819782 10.1016/s0378-5122(98)00039-5

[67] Kravitz HM, et al Sleep difficulty in women at midlife: a community survey of sleep and the menopausal transition. Menopause. 2003;10(1):19–28. 12544673 10.1097/00042192-200310010-00005

[68] Thurston RC, et al Hot flashes and awakenings among midlife women. Sleep. 2019;42(9). doi:10.1093/sleep/zsz131. PMC7368339 31152182

[69] Pien GW, et al Predictors of sleep quality in women in the menopausal transition. Sleep. 2008;31(7):991–999. doi:10.5665/sleep/31.7.991. 18652094 PMC2491505

[70] Ohayon MM, et al Meta-analysis of quantitative sleep parameters from childhood to old age in healthy individuals: developing normative sleep values across the human lifespan. Sleep. 2004;27(7):1255–1273. doi:10.1093/sleep/27.7.1255. 15586779

[71] van den Berg JF, et al Sex differences in subjective and actigraphic sleep measures: a population-based study of elderly persons. Sleep. 2009;32(10):1367–1375. doi:10.1093/sleep/32.10.1367. 19848365 PMC2753814

[72] Chen X, et al Racial/ethnic differences in sleep disturbances: the Multi-Ethnic Study of Atherosclerosis (MESA). Sleep. 2015;38(6):877–888. doi:10.5665/sleep.4732. 25409106 PMC4434554

[73] Cellini N, et al Direct comparison of two actigraphy devices with polysomnographically recorded naps in healthy young adults. Chronobiol Int. 2013;30:691–698. 23721120 10.3109/07420528.2013.782312

