# Supplementary material for: Comparing polysomnography, actigraphy, and sleep diary in the home environment: The Study of Women’s Health Across the Nation (SWAN) Sleep Study
Source: Sleep Adv. 2022 Feb 19;3(1):zpac001. doi: 10.1093/sleepadvances/zpac001 (PMC8918428; doi:10.1093/sleepadvances/zpac001)

Comparing Polysomnography, Actigraphy, and Sleep Diary in the Home Environment: The Study of Women's Health Across the Nation Sleep Study

H. Matthew Lehrer, PhD[1]; Zhigang Yao, PhD[2]; Robert T. Krafty, PhD[3]; Marissa A. Evans, MS[4]; Daniel J. Buysse, MD[1]; Howard M. Kravitz, DO, MPH[5]; Karen A. Matthews, PhD[1]; Ellen B. Gold, PhD[6]; Sioban D. Harlow, PhD[7]; Laura B. Samuelsson, MS[4]; Martica H. Hall, PhD[1]

[1]Department of Psychiatry, University of Pittsburgh, Pittsburgh, PA; [2]Department of Statistics and Applied Probability, National University of Singapore, Singapore; [3]Deparment of Biostatistics and Bioinformatics, Emory University, Atlanta, GA; [4]Department of Psychology, University of Pittsburgh, Pittsburgh, PA; [5]Department of Psychiatry and Behavioral Sciences and Department of Preventive Medicine, Rush University Medical Center, Chicago, IL; [6]Department of Public Health Sciences, University of California, Davis School of Medicine, Davis, CA; [7]Department of Epidemiology, University of Michigan, Ann Arbor, MI

Corresponding Author: Martica H. Hall, PhD, University of Pittsburgh, 3811 O'Hara Street, Room E-1131, Pittsburgh, PA 15213; Phone: 412-246-6431; Fax: 412-246-5300; Email: hallmh@upmc.edu.

Supplementary Figure 1A-E. Residual and model fit statistics for each sleep outcome.

Figure 1A. Residual-based Model Statistics for Time in Bed (TIB).

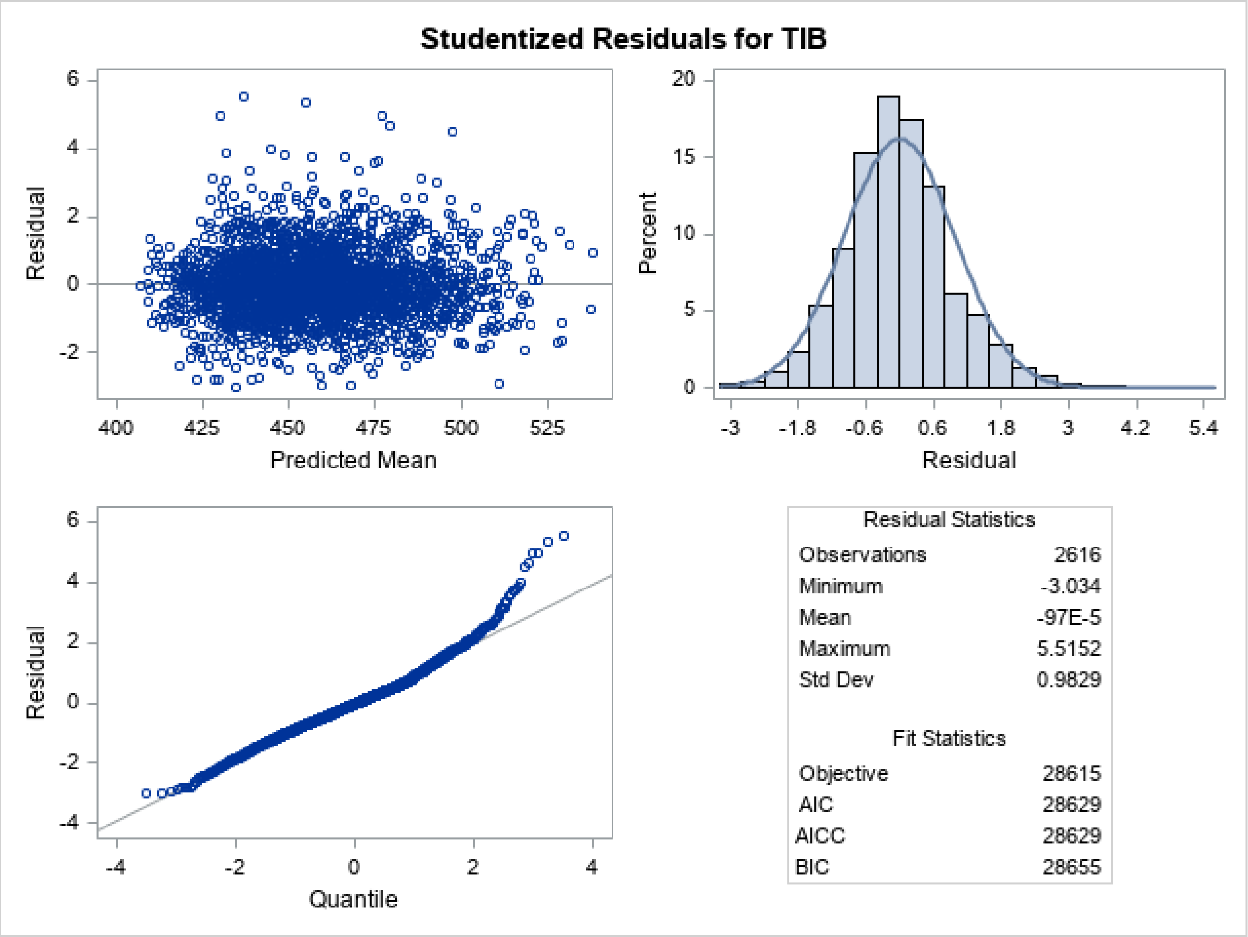

Figure 1B. Residual-based Model Statistics for Total Sleep Time (TST).

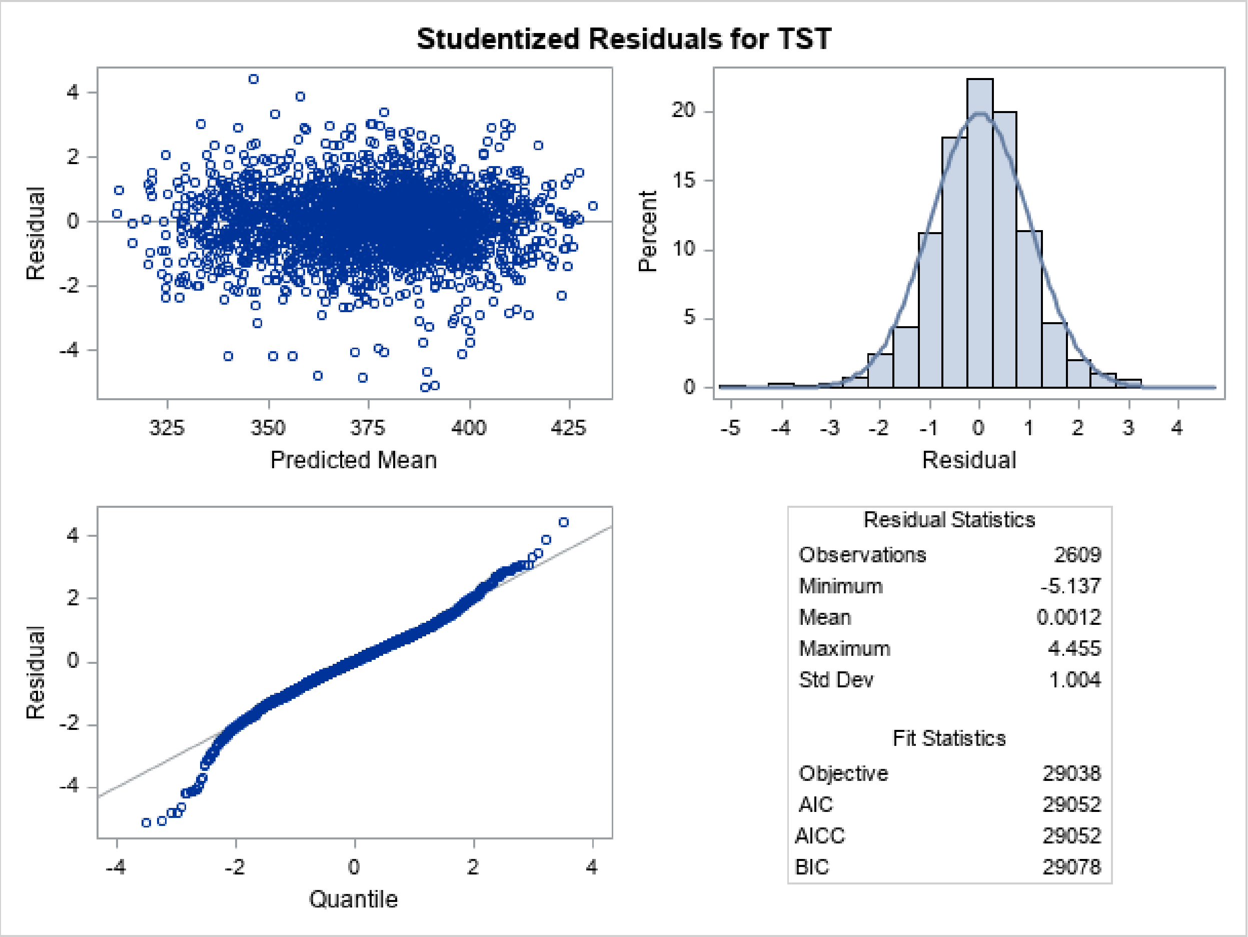

Figure 1C. Residual-based Model Statistics for Sleep Latency (SL).

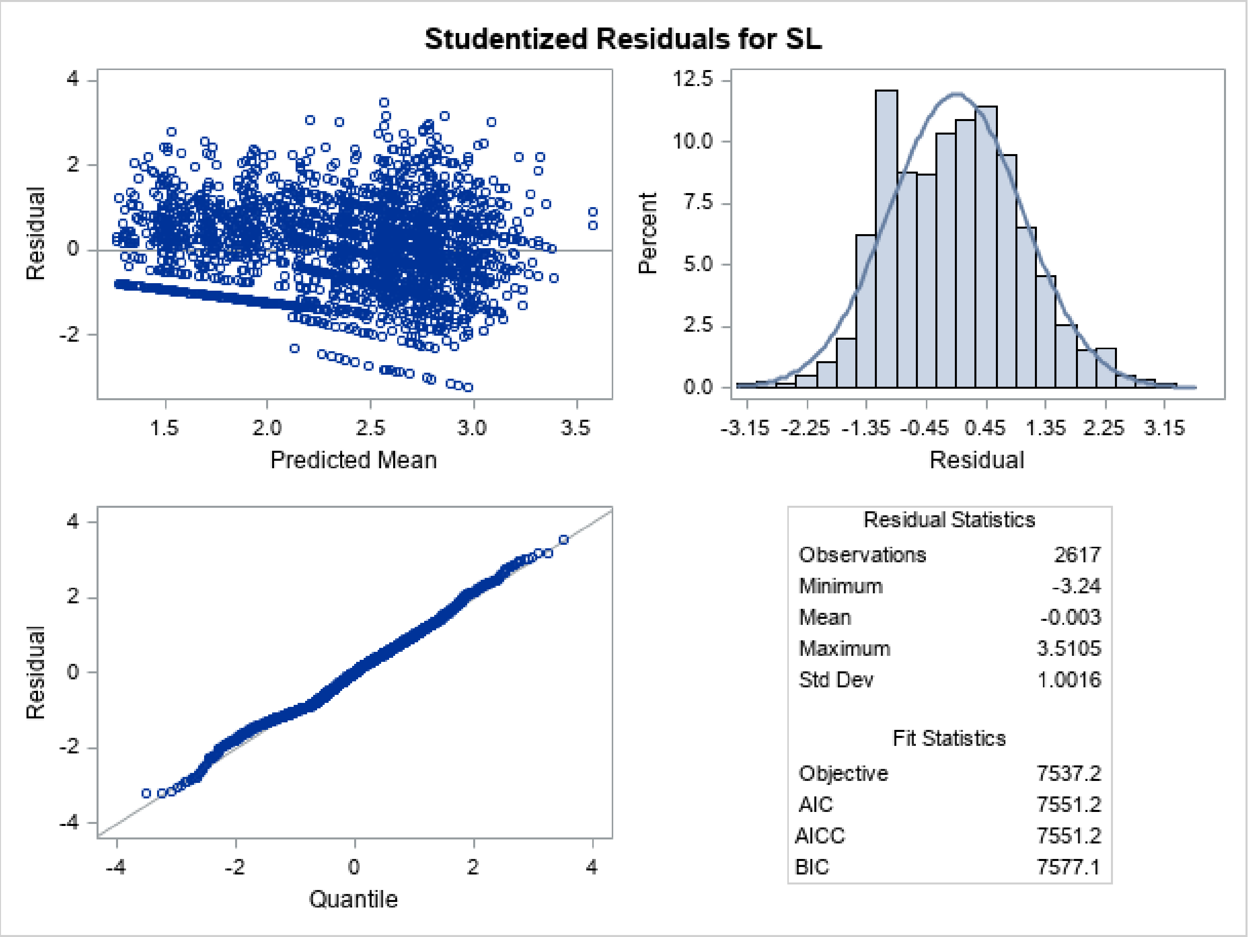

Figure 1D. Residual-based Model Statistics for Wake After Sleep Onset (WASO).

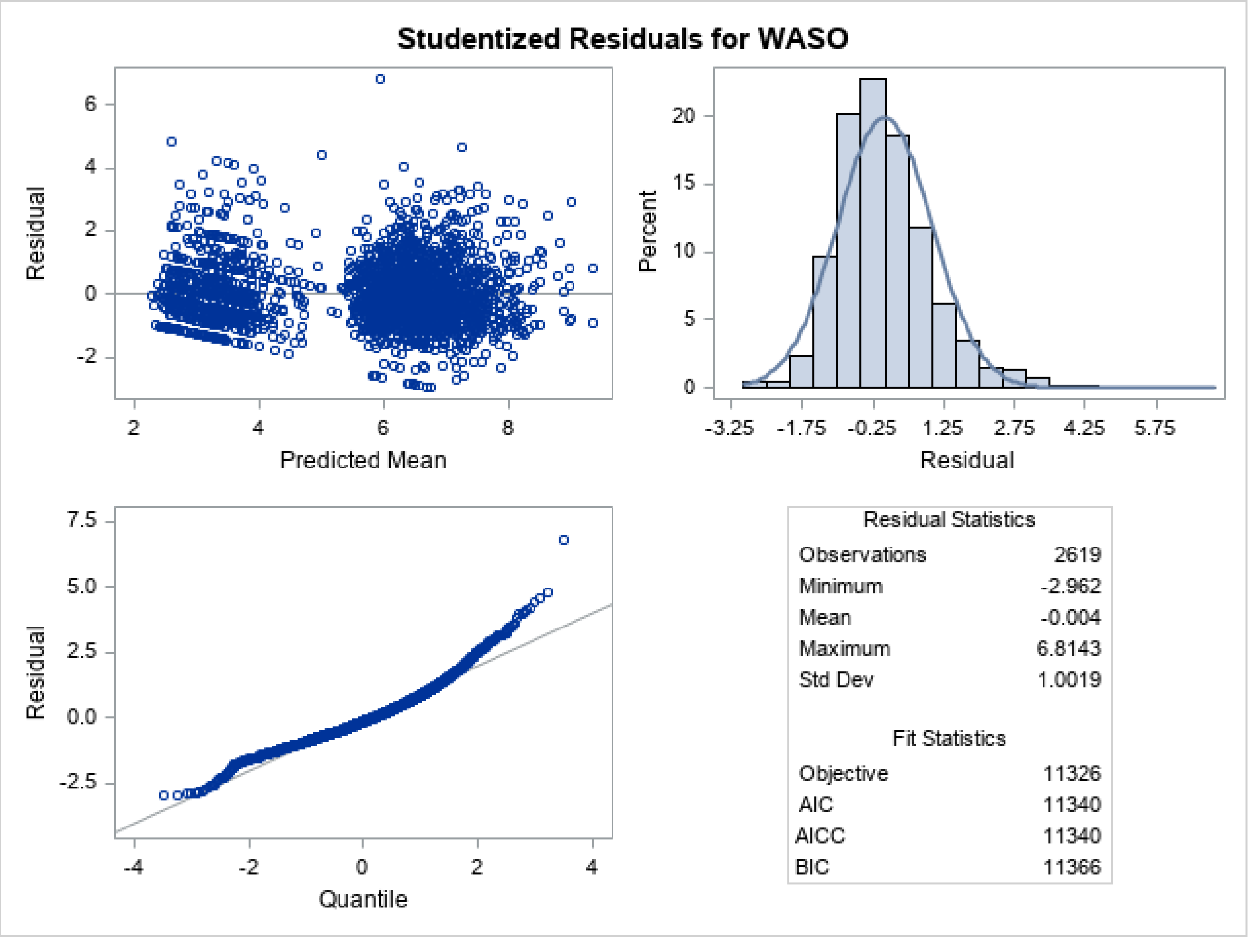

Figure 1E. Residual-based Model Statistics for Sleep Efficiency (SE).

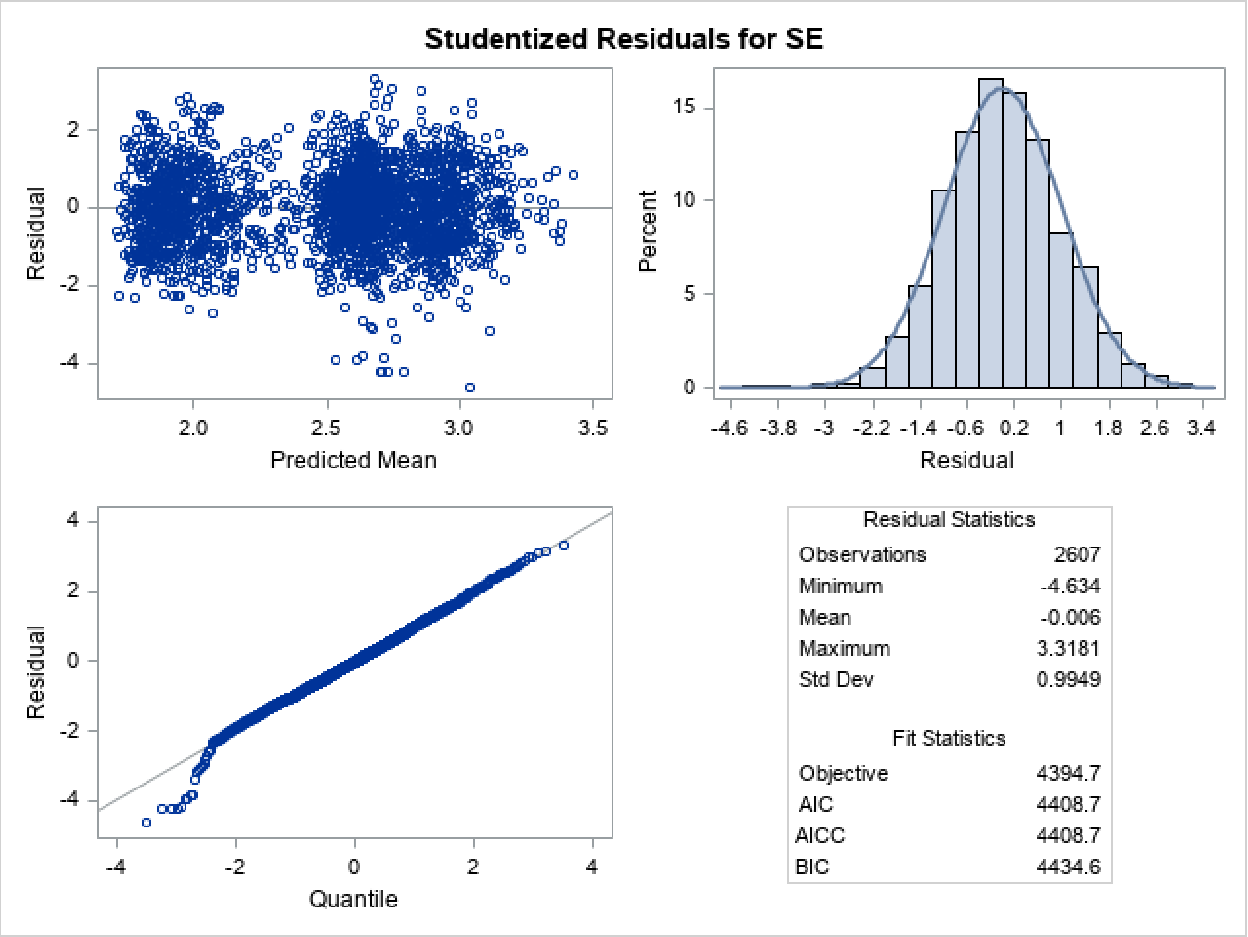

Supplement: zpac001_suppl_Supplementary_Material [file zpac001_suppl_supplementary_material.pdf]
